# Intergenerational relationship quality, sense of loneliness, and attitude toward later life among aging Chinese adults in Hong Kong

**DOI:** 10.3389/fpsyg.2022.930857

**Published:** 2022-08-09

**Authors:** Chang Liu, Shuai Zhou, Xue Bai

**Affiliations:** ^1^Department of Applied Social Sciences, The Hong Kong Polytechnic University, Hong Kong, Hong Kong SAR, China; ^2^Institute of Active Ageing, The Hong Kong Polytechnic University, Hong Kong, Hong Kong SAR, China

**Keywords:** attitude toward later life, aging Chinese adults, sense of loneliness, intergenerational relationships, family

## Abstract

A positive attitude toward later life is crucial for wellbeing among older adults. Maintaining a healthy relationship with adult children can help reduce older parents’ sense of loneliness and nurture a positive life attitude. This study aimed to investigate the associations between multidimensional intergenerational relationship quality and attitudes toward later life among aging Chinese adults in Hong Kong and examine the mediating effects of a sense of loneliness. Representative survey data were collected from 801 participants (aged 50 years and over) with at least one adult child. Multiple linear regression was employed to investigate the associations between overall intergenerational relationship quality with a sense of loneliness as well as the attitude toward later life. To examine the mediating effects of a sense of loneliness, causal mediational analyses were performed. Results demonstrated that overall intergenerational relationship quality was positively associated with aging parents’ attitude toward later life, and this relationship could be partially mediated by a sense of loneliness. Among the four subdomains of intergenerational relationship quality, the influences of structural-associational solidarity and intergenerational conflict on attitude toward later life were almost fully mediated by a sense of loneliness, whereas the influences of consensual-normative solidarity and affectual closeness were partially mediated. These findings contributed to an improved understanding of the relationship between intergenerational relationship quality, sense of loneliness, and attitude toward later life, and could inform future policies and service programs that promote aging adults’ social integration and positive aging.

## Introduction

With prolonged life expectancy and an increase in retirement years, maintaining wellbeing in later life has become increasingly crucial. In positive psychology, it is argued that positive emotions and attitudes can sustain individual wellbeing over time (Fredrickson, 2001). For older adults, a positive life attitude is a key indicator of successful aging. The attitude toward later life refers to one’s perceptions and feelings of his/her life in old age and reflects dispositional characteristics, such as optimistic/pessimistic outlooks (Kato et al., 2012, 2016). The attitude toward later life is a bipolar construct that encompasses both positive and negative views of life. A positive attitude toward later life is characterized by “having a sense of purpose-in-life, and generally feeling content or happy” (Chong et al., 2006). With positive later-life attitudes, older adults could build resources to adapt to life changes and enhance their health and quality of life (Diener et al., 2017). Empirical evidence suggests that older adults with positive life attitudes are more likely to prolong employment (Davies et al., 2017), exhibit a higher level of life satisfaction (Liu et al., 2021), have better physical and psychological wellbeing (Chan et al., 2020), and even live a longer life (Diener and Chan, 2011).

In recent years, growing research has investigated the role of family and intergenerational relationships in shaping later life experiences (e.g., Bai, 2019; Bai et al., 2018; Bai and Liu, 2020). According to life course theory and the concept of “linked lives,” dynamics within the family realm play a crucial role in influencing older adults’ experiences in later life (Elder, 1998). Family members can positively affect older adults’ wellbeing by engaging in retirement planning, serving as anchor points, and facilitating adaptation to retirement life (Szinovacz et al., 2012). In addition to the structure of family relationships, increasing attention has been given to the quality of family relationships (Li and Wang, 2021). It is acknowledged that supportive resources and relationships from family could contribute to positive life attitudes (Yeung and Fung, 2007; Nyqvist et al., 2013; Zhang and Silverstein, 2022). Among all the family members, adult children are increasingly thought to be the closest person by older parents (Lyyra et al., 2010). Studies showed that older parents value their relationship with children and many, particularly older women, expressed the willingness to continue their parental role of supporting children (Nuttman-Shwartz, 2007, 2008; Berkovitch and Manor, 2019). Prior studies also found that positive and strong intergenerational relationships can reduce fearful feelings toward retirement (Sherry et al., 2017) and increase subjective wellbeing (Katz, 2009). It is thus expected that attitude toward later life could be better disentangled through the investigation into the quality of intergenerational relationships with children.

Notably, when examining the influences of intergenerational relationships on later life wellbeing, previous studies mostly treated intergenerational relationship quality as a unidimensional construct and mainly focused on the positive aspect (e.g., Seltzer and Bianchi, 2013). According to the solidarity and conflict models (Bengtson and Roberts, 1991; Clarke et al., 1999), intergenerational relationships have been conceptualized as a multidimensional construct comprising affectual (i.e., positive sentiments and reciprocity of sentiments), consensual (i.e., agreement on values, attitudes, and beliefs), normative (i.e., commitment to performing familial roles and to familial obligation), associational (i.e., interaction in family activities), functional (i.e., exchange of resources), structural (i.e., interaction opportunities), and conflictual (i.e., disagreement and tension) dimensions. Further, based on the solidarity, conflict, and ambivalence models, Bai (2018) has identified a unique four-factor (i.e., affectual closeness, structural-associational solidarity, consensual-normative solidarity, and intergenerational conflict) structure of intergenerational relationship quality that applies to Chinese families. Guided by this theory-guided multidimensional conceptualization (Bai, 2018), this study examined the associations between different domains of intergenerational relationships and attitudes toward later life among aging Chinese parents in Hong Kong.

Furthermore, previous studies have found that the quality of intergenerational relationships as indicated by intergenerational co-residence, contact frequency, and support exchange with children may reduce parents’ perceived isolation or loneliness (Iecovich et al., 2004; de Jong Gierveld and Dykstra, 2008; de Jong Gierveld et al., 2012; Chen and Feeley, 2014; Rodrigues et al., 2014). This is because adult children are the core social network members that remain stable during the aging process and can provide instrumental and emotional support. Under circumstances of family dysfunction, however, loneliness usually arises from losing contact or having poor relationships with family members (Perlman and Peplau, 1981; de Jong Gierveld et al., 2018). Strain from children and ambivalent intergenerational feelings can escalate older parents’ loneliness (Chen and Feeley, 2014; Hua et al., 2021), possibly because that strained or disappointing relationship with children manifests older parents’ unfulfilled generational expectations and thus intensifies loneliness (Tiilikainen and Seppänen, 2017).

Recent evidence shows that the prevalence of loneliness among older adults is surging globally (Luo and Waite, 2014; Wu, 2020). It has been considered a major public health risk for older adults, especially in the context of COVID-19. As a subjective feeling of social isolation, loneliness can arouse an individual’s self-preservation, which in turn leads to a series of psychosocial consequences (Cacioppo and Cacioppo, 2014). A recent review has also shown that being alone or lack of social integration with family and friends generates negative effects on older adults’ wellbeing and social opportunities (Burholt et al., 2020). Hence, it could be gauged that the effect of intergenerational relationship quality on attitude toward later life is partially operated through the sense of loneliness. Despite increasing efforts in exploring the role of family and social integration in later life, the question regarding whether and how a sense of loneliness acts in the association between multidimensional intergenerational relationship quality and attitude toward later life remains to be answered.

### The present study

In Hong Kong, sociocultural and demographic changes during the past decades have challenged the traditional expectations that older people always enjoy harmonious intergenerational relationships (Bai et al., 2018). The proportion of older adults living with children dropped from 53.4% in 2006 to 48.5% in 2016, whereas the proportion of older adults living alone increased from 11.6 to 13.1% (Census and Statistics Department, 2016). Rising education attainment of younger generations and intensified working and living pressure have also challenged traditional values and practice of filial piety and led to increasing heterogeneity in intergenerational relationships in Chinese society. Moreover, recent social movements may have given rise to more intergenerational conflicts between younger generations and their parents (Shek, 2020). Because of the increasingly diverse intergenerational relationships among Hong Kong families, its association with older parents’ attitudes toward later life merits attention and investigation.

Drawing on the four-factor intergenerational relationship model (Bai, 2018), the life course perspective, and the empirical evidence, the present study examined how multiple domains of intergenerational relationship quality are associated with the attitude toward later life through a sense of loneliness, by using a representative sample of aging parents in Hong Kong. Two hypotheses were proposed below:

**Hypothesis 1:** Intergenerational relationship quality is positively associated with the attitude toward later life.

**Hypothesis 2:** The effects of overall intergenerational relationship quality and its four subdomains on the attitude toward later life are mediated by the sense of loneliness.

## Materials and methods

### Participants

Data for the present study were drawn from a citywide representative household survey “Intergenerational Relationship Quality and Care Expectations of Aging Parents in Hong Kong.” The inclusion criteria were specified to recruit Chinese adults (a) aged 50 years or older, (b) residing in Hong Kong, and (c) speaking Cantonese or Mandarin fluently. A standard sampling list was solicited from the Hong Kong Census and Statistics Department. The survey adopted a two-stage stratified random sampling approach. Because the sampling frame covered the Register of Quarters and Register of Segments, the records were initially stratified by geographical region and type of quarters. Using a systematic replicated sampling technique, a random sample of 5,000 addresses was retrieved. Trained interviewers then visited each selected household and used the earliest birthday method to randomly select one eligible participant to conduct the face-to-face questionnaire survey. With the exclusion of inaccessible addresses and households dwelling with no eligible participants, 1,966 respondents were approached and 1,001 aging adults completed the questionnaire. In this study, data analysis was based on the final sample of 801 respondents with at least one adult child.

### Data collection

During November 2016 and March 2017, face-to-face interviews using structural questionnaires were carried out by trained professional interviewers. To minimize the interviewer bias and entry errors, household questionnaire interviews were facilitated by computer-assisted personal interviewing with a web support system. The average duration of each interview was around 40 min. Invitation letters were mailed to the sampled household addresses. Before the interviews, informed consent forms were obtained from respondents. Amongst 1,966 approachable cases, 1,001 respondents participated in the interviews, resulting in a respondent rate of 50.92%. Ethical approval was obtained from the Hong Kong Polytechnic University’s Human Subjects Ethics Sub-Committee.

### Measurements

#### Intergenerational relationship quality

This study used the 13-item Intergenerational Relationship Quality Scale for Aging Chinese Parents (Bai, 2018) to evaluate respondents’ relationships with adult children from four domains: (a) structural-associational solidarity; (b) consensual-normative solidarity; (c) affectual closeness; and (d) intergenerational conflict. Structural-associational solidarity was assessed using four items about residential proximity, contact frequency, face-to-face interaction frequency, and house chores help (e.g., “How often have you contacted each other by phone, letter, or email in the past 12 months?”). Consensual-normative solidarity focused on the similarities in attitudes and values regarding overall opinions, opinions on social issues, and opinions on care responsibility (e.g., “How similar are your opinions on social issues?”). Three questions for affectual closeness asked respondents to rate their general feelings of closeness with, the extent of getting along with, and the frequency of receiving gifts or money from adult children (e.g., “What are your general feelings of closeness to him/her?”). The intergenerational conflict was measured by three items asking questions about the frequency of having tense and strained feelings with, being criticized by, and excessively demanded by adult children (e.g., “How often do you have tense and strained feelings toward him/her?”). All responses were made on five-point Likert scales from 1 to 5. With three items for the intergenerational conflict being reversed, the total score ranged from 13 to 65. For aging parents with more than one child, the average score of the quality of intergenerational relationships with all children was calculated. The reliability coefficient of the scale was 0.776.

#### Sense of loneliness

Participants’ sense of loneliness was assessed by the Chinese version of the De Jong Gierveld Six-item Loneliness Scale (de Jong Gierveld and van Tilburg, 2006; Leung et al., 2008). Unlike other assessment tools focusing on the general state of loneliness, this scale was developed to capture the multidimensionality of loneliness, with three items for emotional loneliness (e.g., “I experience a general sense of emptiness”) and the other three for social loneliness (e.g., “There are many people I can trust completely”). Response categories included “Yes,” “More or Less,” and “No.” All items were recoded into dummy variables (1 = Yes/More or Less, 0 = No). The items for social loneliness were reverse coded. The total scores ranged from 0 to 6, with a higher score indicating a stronger sense of loneliness. The reliability coefficient of the total scale was 0.742.

#### Attitude toward later life

Participants’ attitude toward later life was assessed using the Attitudes Toward Retirement Scale (Atchley and Robinson, 1982). To examine their perceptions about and attitude toward current/future retirement life, participants were asked to rate 14 pairs of bipolar adjectives (e.g., happy–unhappy, meaningful–meaningless, relaxed–tense, and sick–healthy) on a seven-point semantic differential scale. The total scores range from 14 to 98, with a higher score indicating a more positive attitude toward later life. The scale demonstrated high internal consistency (Cronbach’s alpha = 0.952) in our sample.

#### Sociodemographic and health characteristics

The study controlled an array of potential confounders, including demographic characteristics, socioeconomic status, family structure, and health situations. Demographic characteristics included age and gender (0 = female; 1 = male). Socioeconomic status were measured by education (illiterate = 1, elementary school = 2, middle school or higher = 3), employment status (0 = in part- or full-time employment; 1 = retired or no longer working), and economic status. Respondents’ self-reported economic status, ranging from 1 (very strained) to 5 (very rich), was controlled. Family structure was assessed by marital status (1 = married; 0 = unpartnered if the respondent was separated, widowed, or never married) and number of children. In addition, two indicators of health situations were included. General health was assessed by self-rated health status ranging from 1 (very poor) to 5 (very good). Functional health status was assessed by the Lawton Instrumental Activities of Daily Living Scale (IADL; Lawton and Brody, 1969). The aggregate score of IADL was a continuous variable, ranging from 0 to 8. A dummy variable was created to indicate functional dependence (1 = Dependent if having difficulty in at least one aspect of IADL; 0 = Independent).

### Data analysis

The open-source software R (R Core Team, 2021) was used for data analyses. Descriptive analyses were performed to explore the characteristics of key variables. Pairwise correlations between intergenerational relationship quality, sense of loneliness, and attitude toward later life were examined. Following Baron and Kenny’s (1986) classic approach for mediation, models for attitude toward later life and sense of loneliness were fitted separately using multiple linear regression to examine the effects of intergenerational relationship quality. The mediation effects of a sense of loneliness in the association between intergenerational relationship quality together with its subdomains and attitude toward later life were estimated using the *mediation* package (Tingley et al., 2014). The average mediation effects (ACME), average direct effects (ADE), and total effect were reported with quasi-Bayesian confidence intervals with 1,000 simulations. All regression models adjusted for age, gender, education, employment status, economic status, marital status, number of children, self-rated health, and functional health. Influential outliers were checked using leverage and Cook’s distance and excluded from regression models (*n* = 56). The outcome model showed no presence of significant multicollinearity (mean variance inflation factor = 1.669) and heteroscedasticity (Breusch-Pagan statistic = 20.120, *p* = 0.065) issues.

## Results

### Descriptive statistics

Table 1 presents the participants’ socio-demographic characteristics and statistics of all variables. With a mean age of 68.58 years, 56.55% of the participants were female. About 20.90% did not have any formal education, 43.20% finished elementary school, and 35.89% had a middle or higher level of education. Regarding employment status, 25.33% were retired or no longer working. The average self-perceived economic status was 2.95 (SD = 0.60). More than half (59.18%) of participants were in marriage. On average, the number of children of each participant was 2.49. The proportion of respondents with IADL-related difficulties was less than 20%. The mean level of self-rated health was 3.26 (SD = 0.71). Participants scored an average of 44.63 (SD = 6.79) in intergenerational relationship quality. The average score for a sense of loneliness was 2.68 (SD = 1.87). The mean score of the attitude toward later life was 70.99 (SD = 14.25), indicating a generally positive attitude toward later life.

**TABLE 1:** Descriptive statistics.

Variable	Mean	SD	Min	Max
**Age**	68.58	10.88	50	102
**Gender**				
Female (n, %)	453	56.55		
Male (n, %)	348	43.45		
**Education (Missing = 7)**				
Illiterate (n, %)	166	20.91		
Elementary school (n, %)	343	43.20		
Middle school or higher (n, %)	285	35.89		
**Employment status**				
Retired (n, %)	203	25.34		
Working (n, %)	598	74.66		
**Economic status**	2.95	0.60	1	5
**Marital status**				
Unpartnered (n, %)	327	40.82		
Married (n, %)	474	59.18		
**Number of children**	2.49	1.40	1	10
**Self-rated health**	3.26	0.71	1	5
**IADL**				
Independent (n, %)	644	80.40		
Dependent (n, %)	157	19.60		
**Intergenerational relationship quality**	44.63	6.79	17	61
Consensual-normative solidarity	8.49	2.38	3	15
Structural-associational solidarity	13.14	3.61	4	20
Affectual closeness	11.01	2.24	3	15
Intergenerational conflict (reversed)	11.98	2.39	4	15
**Sense of loneliness (Missing = 2)**	2.68	1.87	0	6
**Attitude toward later life**	70.99	14.25	20	98

*N* = 801, SD, standard deviation.

Table 2 presents the results of bivariate correlations among key variables. Attitude toward later life was significantly (*p* < 0.001) and positively correlated with intergenerational relationship quality (*r* = 0.36) and its sub-domains (consensual-normative solidarity: *r* = 0.21, structural-associational solidarity: *r* = 0.27, affectual closeness: *r* = 0.27, and reversed intergenerational conflict: *r* = 0.14), but significantly and negatively correlated with sense of loneliness (*r* = −0.43, *p* < 0.001).

**TABLE 2:** Correlation matrix.

Variable	1	2	3	4	5	6	7
1. Attitude toward later life	1						
2. Intergenerational relationship quality	0.36***	1					
3. Consensual-normative solidarity	0.21***	0.59***	1				
4. Structural-associational solidarity	0.27***	0.74***	0.31***	1			
5. Affectual closeness	0.27***	0.62***	0.23***	0.31***	1		
6. Intergenerational conflict (reversed)	0.14***	0.41***	0.08*	−0.05	0.17***	1	
7. Sense of loneliness	−0.43***	−0.45***	−0.18***	−0.22***	−0.44***	−0.33***	1

Spearman’s correlation coefficients are presented, *p < 0.05; ***p < 0.001.

### Results of regression predicting sense of loneliness and attitude toward later life

Two models were fitted to estimate the effects of intergenerational relationship quality on the sense of loneliness and attitude toward later life. Both models were adjusted for demographic characteristics, socioeconomic status, family structure, and health situations. As shown in Table 3, Model 1 explained about 31.7% of variance in sense of loneliness (*F* = 31.97, *p* < 0.001). Model 2 which included intergenerational relationship quality, sense of loneliness, and all covariates contributed to 55.4% of variance explained in attitude toward later life (*F* = 77.083, *p* < 0.001).

**TABLE 3:** Results of multiple linear regression predicting loneliness and attitude toward later life.

	Sense of loneliness	Attitude toward later life
	
	Model 1	Model 2
Age	−0.016^+^ (0.008)	−0.280*** (0.049)
Gender*^a^*	0.331* (0.132)	0.746 (0.769)
Elementary school*^b^*	−0.045 (0.173)	0.401 (1.004)
Middle school or higher*^b^*	−0.192 (0.202)	−0.788 (1.172)
Employment status*^c^*	0.253^+^ (0.152)	−2.651** (0.885)
Economic status	−0.339** (0.110)	4.113*** (0.645)
Marital status*^d^*	−0.547*** (0.127)	0.923 (0.748)
Number of children	−0.293*** (0.048)	−0.309 (0.286)
Self-rated health	−0.321*** (0.092)	4.175*** (0.535)
IADL*^e^*	0.187 (0.177)	−6.591*** (1.028)
Intergenerational relationship quality	−0.117*** (0.010)	0.214*** (0.062)
Sense of loneliness		−1.731*** (0.216)
Constant	11.814*** (0.803)	62.715*** (5.310)
*R* ^2^	0.327	0.562
Adjusted *R*^2^	0.317	0.554
F Statistic	31.971***	77.083***
AIC	2723.015	5308.135
BIC	2782.814	5372.533

*N* = 735, ^*a*^Female = 0, male = 1. ^*b*^Reference group = illiterate. ^*c*^Retired = 0, working = 1. ^*d*^unpartnered = 0, married = 1. ^*e*^IADL was recoded into a dummy variable (0 = Independent, 1 = Dependent). AIC, Akaike’s information criterion; BIC, Bayesian information criterion. Standard errors are in parentheses, ^+^*p* < 0.1; *p < 0.05; **p < 0.01; ***p < 0.001.

In Model 1, the coefficients of intergenerational relationship quality were negative and statistically significant (sense of loneliness: *B* = −0.117, SE = 0.010, *p* < 0.001), with demographic, socioeconomic, family-related, and health covariates being adjusted. The model also revealed that loneliness was more common among those who were male, with lower economic status, unpartnered, raised fewer children, and had poor health.

Model 2 shows that the direct effect of intergenerational relationship quality on attitude toward later life was positive and significant (*B* = 0.214, SE = 0.062, *p* < 0.001), when sense of loneliness was introduced. Additionally, sense of loneliness (*B* = −1.731, SE = 0.216, *p* < 0.001) was negatively associated with attitude toward later life in Model 2. Moreover, the results suggested that participants who were younger, not working, with abundant economic resources, healthy, and functionally independent tended to hold a more positive attitude toward later life.

### Results of causal mediation analyses

Causal mediation analyses were performed to further examine the mediating effects of a sense of loneliness on the association between intergenerational relationship quality and attitude toward later life. Table 4 presents the results of mediation analyses. The proportions of indirect effect on attitude toward later life (Prop. Mediated) through sense of loneliness were presented. In general, the effect of intergenerational relationship quality on later life attitude was partially mediated by both sense of loneliness (ACME = 0.202, ADE = 0.216, Total Effect = 0.418, Prop. Mediated = 48.00%).

**TABLE 4:** Mediating effects of the sense of loneliness.

Independent variable	Mediator	ACME	ADE	Total effect	Prop. mediated
Intergenerational relationship quality	Sense of loneliness	0.202***	0.216***	0.418***	48.00***
Consensual-normative solidarity	Sense of loneliness	0.255***	0.504**	0.758***	33.60***
Structural-associational solidarity	Sense of loneliness	0.220***	0.119	0.340**	Full mediation**
Affectual closeness	Sense of loneliness	0.504***	0.818***	1.322***	38.10***
Intergenerational conflict (reversed)	Sense of loneliness	0.403***	−0.080	0.324*	Full mediation*

*N* = 735, Prop. Mediated, proportions of indirect effect on attitude toward later life via mediators (%). ACME, average mediation effect. ADE, average direct effect. All models adjusted for age, gender, education, employment status, economic status, marital status, number of children, self-rated health, and IADL. Quasi-Bayesian confidence intervals were not reported here, *p < 0.05; **p < 0.01; ***p < 0.001.

**FIGURE 1:**
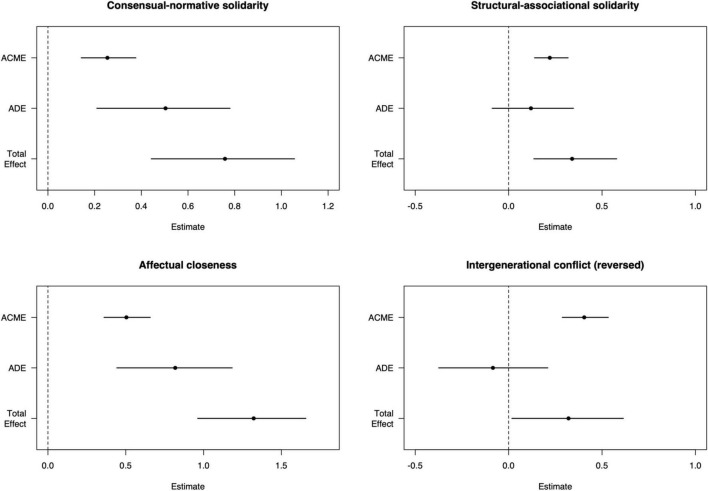
Results of the mediation analyses. Error bar shows 95% quasi-Bayesian confidence intervals. ACME, average mediation effect; ADE, average direct effect.

The total effects of consensual-normative solidarity on attitude toward later life were significant at *p* < 0.001 level and could be partially mediated by sense of loneliness (ACME = 0.255, ADE = 0.504, Total Effect = 0.758, Prop. Mediated = 33.60%; see Figure 1). Similarly, 38.10% of the total effects of affectual closeness were mediated through sense of loneliness (ACME = 0.504, ADE = 0.818, Total Effect = 1.322).

Results suggested that the association between structural-associational solidarity and attitude toward later life was almost fully mediated by sense of loneliness (ACME = 0.220, ADE = 0.119 *n.s*., Total Effect = 0.340). Similarly, loneliness entirely channeled the effect of intergenerational conflict on attitude toward later life because the direct effect disappeared in the outcome model (ACME = 0.403, ADE = −0.080 *n.s.*, Total Effect = 0.324).

### Sensitivity analysis

We performed several sensitivity analyses to check the robustness of the results. First, we examined whether the mediation analysis violated the sequential ignorability assumption (Imai et al., 2010), which requires (a) the independence of error terms in mediator and outcome models and (b) no potential omitted confounders. As shown in Supplementary Figure 1 in the Supplementary material, the estimate of ACME would decrease to zero if the correlation between error terms in the mediator and outcome models (Models 1 and 2 in Table 3) reaches about −0.30. In addition, the estimate of ACME would change sign only if the proportion of total variance in sense of loneliness and attitude toward later life explained by confounders is larger than 0.03. For instance, to invalidate the ACME, a confounder must explain at least 20% of the variance in attitude toward later life and 15% of the variance in sense of loneliness, respectively. It suggests that the indirect effect of intergenerational relationship quality on loneliness through sense of loneliness is moderately robust against the sequential ignorability assumption.

Second, we checked whether the main results were sensitive to age structure. Because our sample included a wide range of older adults, some may concern that the results downplayed the age heterogeneity. It is possible that Chinese parents at older ages may have a higher reliance on adult children for care and support than their younger counterparts. We stratified the analysis by age group. As shown in Supplementary Tables 1, 2, the mediating effect of sense of loneliness strongly persisted with age, although the proportion of indirect effect decreased in older age groups. The association between intergenerational relationship quality and attitude toward later life was moderately mediated by loneliness in both the older (65–79 years) and oldest (aged 80 years and above) groups. Among younger respondents aged 50 to 64 years, the association between intergenerational relationship quality and attitude toward later life was almost fully mediated by sense of loneliness.

Third, we further evaluated the influence of outliers on our results (Supplementary Table 3). It showed that the associations of intergenerational relationship quality with loneliness and attitude toward later life were robust against outliers.

## Discussion and implications

Based on representative survey data collected in Hong Kong, this study examined the direct association between intergenerational relationship quality and attitude toward later life and their indirect associations through the sense of loneliness. Our results demonstrated that the quality of intergenerational relationships and the four subdomains (i.e., consensual-normative solidarity, structural-associational solidarity, affectual closeness, and the reverse-coded intergenerational conflict) were positively associated with aging adults’ attitudes toward later life. Meanwhile, the sense of loneliness partially mediated the effects of overall intergenerational relationship quality on attitude toward later life. Among the four subdomains of intergenerational relationship quality, the effects of structural-associational solidarity and intergenerational conflict on attitude toward later life were almost fully mediated by sense of loneliness, whereas the effects of consensual-normative solidarity and affectual closeness were only partially mediated.

The findings of this study supported that intergenerational relationship quality was significantly associated with aging parents’ attitudes toward later life. It is possible that because adult children can provide support during the aging and retirement transition process of their aging parents, thereby reducing parents’ fear of future life and emotional distress (Sherry et al., 2017). With close and secure relationships with children, aging parents thus hold a more optimistic view on retirement life. This is also consistent with previous qualitative findings that suggested that the meaning of later life for aging parents is partly defined by the opportunity to continue or strengthen their parental roles (Nuttman-Shwartz, 2007, 2008; Berkovitch and Manor, 2019). In terms of its subdomains, our study found that the aspects of intergenerational solidarity, including consensual-normative solidarity, structural-associational solidarity, and affectual closeness, were associated with a higher level of optimistic attitude toward later life, while the conflictual aspect was associated with an elevated pessimistic attitude toward later life. The different effects of the various aspects of intergenerational relationship quality confirmed the necessity of treating it as a multidimensional phenomenon. The attitude toward later life can be promoted not only by facilitating intergenerational solidarity but also by preventing and managing intergenerational conflicts.

Current research findings indicated that positive intergenerational relationship quality could reduce the level of loneliness, whereas negative relationship quality such as intergenerational conflict (not reversed) exacerbates loneliness. This was also reflected in a previous study which found that greater intergenerational ambivalence was related to increased loneliness among older adults (Hua et al., 2021). Given the crucial role of intergenerational relationships in promoting adaptations in later life, policies and social service programs should be developed to cultivate supportive and harmonious relationships between adult children and older parents, facilitate intergenerational communication and mutual understanding, and deliver strategies to manage intergenerational conflict.

Notably, this study revealed that the association between intergenerational relationship quality and attitude toward later life could be partially mediated *via* the sense of loneliness. A possible explanation might be that under continuous impacts of sociocultural transformation, aging parents who had weakened functional support from children may be more often to be socially isolated and lonely. The results verified Hypothesis 2 and further revealed that the effects of structural-associational solidarity as well as intergenerational conflict on attitude toward later life were almost completely mediated by the sense of loneliness, but the effects of the remaining two subdomains were partially mediated. It suggests that loneliness is an important intermediate factor that largely explains negative life attitudes due to reduced contact and increased strain feelings between older parents and adult children. Such findings imply a direction that interventions targeting retirees from a family perspective should pour efforts into solving loneliness among those living without children in proximity and having conflictual intergenerational relationships. In view of this, to promote later life wellbeing among older adults, social policies and services should pay more attention to their social integration, helping them broaden social participation and expand peer networks, especially for those with poor family relationships. In addition, our sensitivity analysis showed that the mediating effect of sense of loneliness was largest among younger parents (aged 50–64 years) and smallest among oldest parents (aged 80 years and above). The findings may highlight the aged-related variation in response to changes in the quality of intergenerational relationships. When reaching advanced ages, older adults’ relationships with children might have a strong and direct effect on their attitude toward later life. Hence, tailored interventions that promote intergenerational solidarity should attend to this age pattern to effectively alleviate loneliness and foster positive life attitudes for older adults.

### Limitations and future research

Despite its contributions, some limitations of this study need to be acknowledged. First, this study used a cross-sectional design, so the interpretation of the results should be considered with some caution. To deepen the understanding of causal relationships, researchers could adopt a longitudinal design to trace changes in intergenerational relationships, loneliness, and later-life attitudes. Second, our analysis of the association between intergenerational relationships and attitudes toward later life cannot capture the impacts of recent societal changes. There is concern that intergenerational conflicts have been aggravated in Hong Kong Chinese families due to the social unrest during 2019 to 2020 (Shek, 2020). The social distancing measures, including lockdown, during the COVID-19 pandemic have also increased many older adults’ social isolation and loneliness. Future research may further explore how intergenerational relationships and loneliness affect the later-life attitudes of older adults in the post-pandemic era. Finally, this research only focused on the role of intergenerational relationships in the family context. Future research could compare intergenerational relationships with sibling and marital relationships in facilitating positive aging and explore the socio-demographic variation in intergenerational relationships.

## Conclusion

This study unveiled the association between multi-dimensional intergenerational relationship quality and attitude toward later life using a representative sample of aging adult parents in Hong Kong and supported the mediating effects of the sense of loneliness. Among the four subdomains of intergenerational relationship quality, the influences of structural-associational solidarity and intergenerational conflict on attitude toward later life were almost fully mediated by sense of loneliness, whereas the influences of consensual-normative solidarity and affectual closeness were partially mediated. Our sensitivity analyses supported the robustness of the results and revealed the age-related heterogeneity in the mediating roles of sense of loneliness. The findings have implications for developing policies and service programs to promote intergenerational solidarity and social integration for aging adults.

## Data availability statement

The original contributions presented in this study are included in the article/Supplementary material, further inquiries can be directed to the corresponding author.

## Ethics statement

The studies involving human participants were reviewed and approved by the Human Subjects Ethics Sub-Committee at The Hong Kong Polytechnic University. The patients/participants provided their written informed consent to participate in this study.

## Author contributions

CL contributed to the study design and manuscript revision. SZ did the statistical analyses and wrote the manuscript. XB contributed to the study conception and manuscript revision. All authors contributed to the article and approved the submitted version.
