# Supplementary material for: Intergenerational relationship quality, sense of loneliness, and attitude toward later life among aging Chinese adults in Hong Kong
Source: Front Psychol. 2022 Aug 9;13:930857. doi: 10.3389/fpsyg.2022.930857 (PMC9397484; doi:10.3389/fpsyg.2022.930857)
Supplement: Supplementary file 1 [file Data_Sheet_1.docx]

Supplementary Material

**1 Sensitivity analysis: Sequential ignorability assumption**


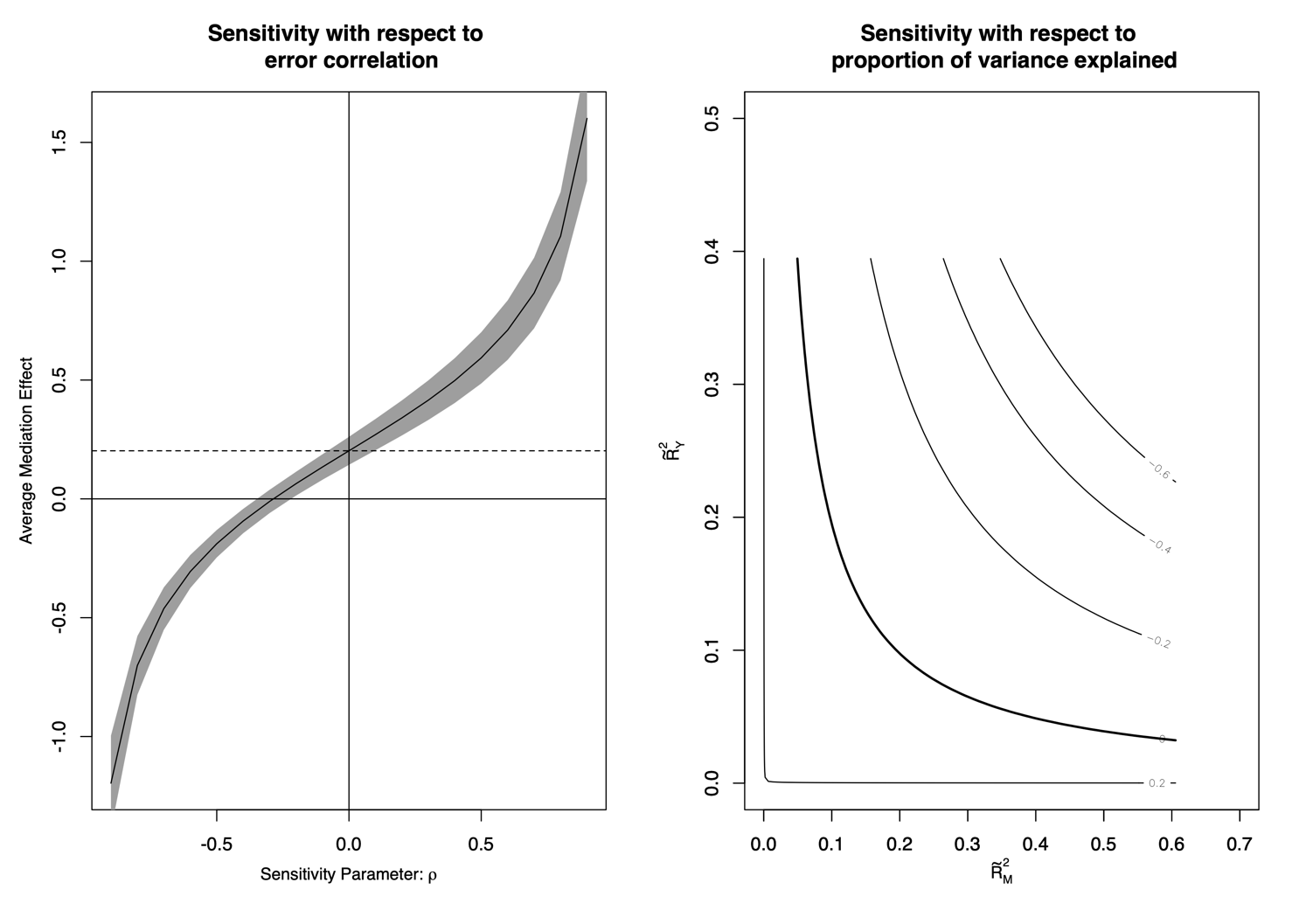


**Figure 1.** Sensitivity analysis for ACME. The left panel depicts the estimated ACME at different levels of the sensitivity parameter $\rho$ (the correlation between residuals in mediator and outcome models, namely Models 1 for sense of loneliness and 2 for attitude toward later life in Table 3 of the article). The dark line and shadow area represent the estimates of ACME with the 95% confidence intervals estimated via 1,000 simulations. The dashed line shows the true value of ACME (Estimate = 0.202, *p* < 0.001). The right panel depicts the estimates of ACME as a function of the explained proportions of variance in the mediator ($\tilde{R}_{M}^{2}$, horizontal axis) and outcome ($\tilde{R}_{Y}^{2}$, vertical axis) by unobserved omitted confounders. The confounders are assumed to be associated with the mediator and outcome variable in different directions. The contour line denotes the ACME at specific values of $\tilde{R}_{M}^{2}$ and $\tilde{R}_{Y}^{2}$.

**2 Sensitivity analysis: Stratified analysis**

In Table 1, we stratified the sample by age. Models 1 and 2 estimated the effect of intergenerational relationship quality on the attitude toward retirement life among the younger group (aged 50-64). The results suggest that the direct effect of intergenerational relationship quality on attitude toward later life was significant (B = 0.119, *p* > 0.05) after controlling loneliness. In Models 3 and 4, we conducted the analyses using an older sample (aged 65-79). We found that the direct effect of intergenerational relationship quality on attitude toward later life remained significant (B = 0.196, *p* < 0.05). In the last two columns, the models for oldest adults (aged 80 and above) show a significant effect of intergenerational relationship quality on attitude toward later life (B = 0.319, *p* < 0.05). Intergenerational relationship quality was negatively and significantly associated with sense of loneliness in three age groups. It may indicate that loneliness might fully mediate the association between intergenerational relationship quality and attitude toward later life for the younger group, but partially mediating such association for older and oldest groups. According to the goodness-of-fit indices, the model explained a larger proportion of variance in both the sense of loneliness (about 40%) and attitude toward later life (about 55%) among the oldest group.

**Table 1.** **Results of multiple linear regression predicting attitude toward later life in different age groups.**

|  | **Younger group**  **(Aged 50-64)** | | **Older group**  **(Aged 65-79)** | | **Oldest group**  **(Aged 80 and above)** | |
| --- | --- | --- | --- | --- | --- | --- |
|  | **Sense of loneliness** | **Attitude toward later life** | **Sense of loneliness** | **Attitude toward later life** | **Sense of loneliness** | **Attitude toward later life** |
|  | **Model 1** | **Model 2** | **Model 3** | **Model 4** | **Model 5** | **Model 6** |
| Age | 0.001 (0.025) | 0.167 (0.149) | -0.051^*^ (0.024) | -0.138 (0.135) | 0.015 (0.035) | -0.721^***^ (0.194) |
| Gender ^a^ | 0.321 (0.222) | 0.197 (1.336) | 0.284 (0.200) | 1.142 (1.121) | 0.319 (0.318) | 0.958 (1.767) |
| Elementary school ^b^ | -0.723 (0.472) | -0.292 (2.843) | -0.077 (0.252) | -0.640 (1.410) | 0.309 (0.326) | 0.848 (1.808) |
| Middle school or higher ^b^ | -0.902^+^ (0.468) | -1.210 (2.823) | -0.086 (0.301) | -1.144 (1.682) | -0.417 (0.516) | -4.297 (2.861) |
| Employment status ^c^ | 0.173 (0.203) | -2.483^*^ (1.221) | 0.469^+^ (0.284) | -4.076^*^ (1.595) | 1.759 (1.173) | -6.994 (6.549) |
| Economic status | -0.219 (0.187) | 2.760^*^ (1.125) | -0.305^+^ (0.170) | 5.536^***^ (0.955) | -0.841^***^ (0.248) | 2.689^+^ (1.435) |
| Marital status ^d^ | -0.346 (0.216) | 0.948 (1.302) | -0.565^**^ (0.189) | 0.940 (1.071) | -0.768^*^ (0.311) | 2.586 (1.764) |
| Number of children | -0.398^***^ (0.097) | -0.653 (0.598) | -0.281^***^ (0.074) | -0.272 (0.422) | -0.197^*^ (0.090) | 0.248 (0.506) |
| Self-rated health | -0.359^**^ (0.133) | 2.677^**^ (0.809) | -0.455^**^ (0.155) | 5.390^***^ (0.881) | 0.026 (0.229) | 5.507^***^ (1.267) |
| IADL ^e^ | 0.699 (0.782) | -4.952 (4.692) | 0.347 (0.251) | -5.641^***^ (1.405) | -0.214 (0.291) | -6.606^***^ (1.616) |
| Intergenerational relationship quality | -0.124^***^ (0.016) | 0.119 (0.106) | -0.109^***^ (0.015) | 0.196^*^ (0.091) | -0.130^***^ (0.022) | 0.319^*^ (0.138) |
| Sense of loneliness |  | -2.287^***^ (0.357) |  | -1.377^***^ (0.325) |  | -1.392^**^ (0.503) |
| Constant | 11.674^***^ (1.730) | 53.609^***^ (11.175) | 14.041^***^ (1.904) | 45.491^***^ (11.581) | 8.563^*^ (3.319) | 96.701^***^ (18.856) |
| *N* | 294 | 294 | 308 | 308 | 133 | 133 |
| R^2^ | 0.301 | 0.316 | 0.323 | 0.493 | 0.453 | 0.590 |
| Adjusted R^2^ | 0.274 | 0.287 | 0.298 | 0.473 | 0.403 | 0.548 |
| F Statistic | 11.039^***^ | 10.813^***^ | 12.823^***^ | 23.950^***^ | 9.112^***^ | 14.361^***^ |
| AIC | 1094.994 | 2149.018 | 1156.092 | 2217.212 | 496.844 | 952.715 |
| BIC | 1142.880 | 2200.588 | 1204.583 | 2269.433 | 534.418 | 993.180 |

^a^ Female =0, male ==1. ^b^ Reference group = illiterate. ^c^ Retired = 0, working = 1. ^d^ unpartnered = 0, married = 1. ^e^ IADL was recoded into a dummy variable (0 = Independent, 1 = Dependent). AIC, Akaike's information criterion; BIC, Bayesian information criterion. Standard errors are in parentheses.

^+^ *p* < 0.1; ^*^*p* < 0.05; ^**^*p* < 0.01; ^***^ *p* < 0.001.

Table 1 suggests that the effect of sense of loneliness on attitude toward retirement life may be contingent on age. To further explore the mediating role of sense of loneliness, we performed mediational analyses stratified by age group (Table 2). The results indicated that sense of loneliness mediated about nearly all of the total effect of intergenerational relationship quality among the younger group. For those aged 65-79 years, approximately 43% of the total effect of intergenerational relationship quality on attitude toward later life was mediated by sense of loneliness. It also shows that sense of loneliness mediated about 36% of the total effect of intergenerational relationship quality on attitude toward later life in the oldest group.

**Table 2. Mediating effects of sense of loneliness in different age groups.**

| **Model** | **Independent variable** | **Mediator** | **ACME** | **ADE** | **Total Effect** | **Prop. Mediated** |
| --- | --- | --- | --- | --- | --- | --- |
| Younger adults (Aged 50-64, *N* = 294) | Intergenerational relationship quality | Sense of loneliness | 0.278^***^ | 0.125 | 0.403^***^ | 69.30^***^ |
| Older adults (Aged 65-79, *N* = 308) | Intergenerational relationship quality | Sense of loneliness | 0.150^***^ | 0.198^*^ | 0.348^***^ | 42.97^***^ |
| Oldest adults (Aged 80 and above, *N* = 133) | Intergenerational relationship quality | Sense of loneliness | 0.179^**^ | 0.316^*^ | 0.495^***^ | 35.56^**^ |

Prop. Mediated, proportions of indirect effect on attitude toward later life via mediators (%). ACME, average mediation effect. ADE, average direct effect. All models adjusted for age, education, gender, employment status, economic status, marital status, number of children, self-rated health, and IADL. Quasi-Bayesian confidence intervals were not reported here.

^*^*p* < 0.05; ^**^*p* < 0.01; ^***^ *p* < 0.001.

Overall, intergenerational relationship quality was negatively associated with sense of loneliness and positively associated with attitude toward later life. The mediating effects of sense of loneliness were larger in younger age groups (50-64 years) than in older (65-79 years) or oldest (80 years and above) groups. It may indicate an age-related pattern that younger parents who have a lower quality of intergenerational relationships may substantially escalate the feeling of loneliness, but older and oldest parents with lower quality of intergenerational relationships may develop other potential pathways that explain their attitude toward later life.

**2 Sensitivity analysis: The influence of outliers**

We also assessed whether outliers could significantly alter our results. In the table below, we estimated the associations between intergenerational relationship quality with loneliness and attitude toward later life using the sample that included outliers (*N* = 791). In general, the coefficients of intergenerational relationship quality and sense of loneliness were similar to Table 3 of the article, which excluded outliers. Nonetheless, the inclusion of outliers decreased the goodness of fit in modeling the attitude toward later life compared to Table 3 of the article.

**Table 3. Results of multiple linear regression predicting attitude toward later life (With outliers).**

|  | **Sense of loneliness** | **Attitude toward later life** |
| --- | --- | --- |
|  | **Model 1** | **Model 2** |
| Age | -0.013 (0.008) | -0.254^***^ (0.051) |
| Gender ^a^ | 0.318^*^ (0.128) | 0.676 (0.837) |
| Elementary school ^b^ | 0.019 (0.165) | 0.060 (1.078) |
| Middle school or higher ^b^ | -0.138 (0.193) | -0.405 (1.256) |
| Employment status ^c^ | 0.262^+^ (0.150) | -2.826^**^ (0.980) |
| Economic status | -0.339^***^ (0.101) | 3.962^***^ (0.663) |
| Marital status ^d^ | -0.551^***^ (0.125) | 0.055 (0.823) |
| Number of children | -0.246^***^ (0.043) | -0.142 (0.288) |
| Self-rated health | -0.304^***^ (0.085) | 4.559^***^ (0.555) |
| IADL ^e^ | 0.182 (0.167) | -6.654^***^ (1.088) |
| Intergenerational relationship quality | -0.110^***^ (0.009) | 0.186^**^ (0.064) |
| Sense of loneliness |  | -1.857^***^ (0.234) |
| Constant | 11.040^***^ (0.746) | 62.081^***^ (5.503) |
| R^2^ | 0.314 | 0.503 |
| Adjusted R^2^ | 0.304 | 0.495 |
| F Statistic | 32.442^***^ | 65.599^***^ |
| AIC | 2956.081 | 5922.870 |
| BIC | 3016.834 | 5988.297 |

*N* = 791. ^a^ Female =0, male ==1. ^b^ Reference group = illiterate. ^c^ Retired = 0, working = 1. ^d^ unpartnered = 0, married = 1. ^e^ IADL was recoded into a dummy variable (0 = Independent, 1 = Dependent). AIC, Akaike's information criterion; BIC, Bayesian information criterion. Standard errors are in parentheses.

^+^ *p* < 0.1; ^*^*p* < 0.05; ^**^*p* < 0.01; ^***^ *p* < 0.001.
